# Screening of key biomarkers and immune infiltration in Pulmonary Arterial Hypertension via integrated bioinformatics analysis

**DOI:** 10.1080/21655979.2021.1936816

**Published:** 2021-07-07

**Authors:** Yu Zeng, Nanhong Li, Zhenzhen Zheng, Riken Chen, Wang Liu, Junfen Cheng, Jinru Zhu, Mingqing Zeng, Min Peng, Cheng Hong

**Affiliations:** a Department of Respiration, The Second Affiliated Hospital of Guangdong Medical University, Zhanjiang, Guangdong, China; b Institute of Nephrology, Affiliated Hospital of Guangdong Medical University, Zhanjiang, Guangdong, China; c China State Key Laboratory of Respiratory Disease, National Clinical Research Center for Respiratory Disease, the First Affiliated Hospital of Guangzhou Medical University, Guangzhou, Guangdong, China; d First Clinical School of Medicine, Guangdong Medical University, Zhanjiang, Guangdong, China

**Keywords:** Bioinformatics analysis, differentially expressed genes (degs), pulmonary arterial hypertension (pah), immune infiltration, immune system function

## Abstract

This study aimed to screen key biomarkers and investigate immune infiltration in pulmonary arterial hypertension (PAH) based on integrated bioinformatics analysis. The Gene Expression Omnibus (GEO) database was used to download three mRNA expression profiles comprising 91 PAH lung specimens and 49 normal lung specimens. Three mRNA expression datasets were combined, and differentially expressed genes (DEGs) were obtained. Gene ontology (GO) and Kyoto Encyclopedia of Genes and Genomes (KEGG) analyses and the protein-protein interaction (PPI) network of DEGs were performed using the STRING and DAVID databases, respectively. The diagnostic value of hub gene expression in PAH was also analyzed. Finally, the infiltration of immune cells in PAH was analyzed using the CIBERSORT algorithm. Total 182 DEGs (117 upregulated and 65 downregulated) were identified, and 15 hub genes were screened. These 15 hub genes were significantly associated with immune system functions such as myeloid leukocyte migration, neutrophil migration, cell chemotaxis, Toll-like receptor signaling pathway, and NF-κB signaling pathway. A 7-gene-based model was constructed and had a better diagnostic value in identifying PAH tissues compared with normal controls. The immune infiltration profiles of the PAH and normal control samples were significantly different. High proportions of resting NK cells, activated mast cells, monocytes, and neutrophils were found in PAH samples, while high proportions of resting T cells CD4 memory and Macrophages M1 cell were found in normal control samples. Functional enrichment of DEGs and immune infiltration analysis between PAH and normal control samples might help to understand the pathogenesis of PAH.

## Research highlights

1. A 7-gene-based model had better diagnostic value in identifying PAH tissues.

2. The immune infiltration analysis might help understand the pathogenesis of PAH.

3. Bioinformatics provides a new perspective for the study of pathogenesis of PAH.

## 1. Introduction

Pulmonary arterial hypertension (PAH) can be a separate disease or pathophysiological syndrome of abnormally elevated pulmonary artery pressure caused by known or unknown reasons, with a relatively low survival rate [1,2]. The prevalence rate of PAH is 15–50 cases/million people/year, with an incidence rate is 5–10 cases/million people [3]. Untreated pulmonary hypertension patients had an average survival time of about 2.8 years before approximately 40 years ago [4]. Just like the diagnosis and therapy progression of PAH, its mortality rate has greatly improved, although it is still high, with a 5-year survival rate of 61.2% for newly diagnosed PAH patients [5]. Thus, to further search for clinical molecular markers, the pathogenesis and progression of PAH is still an important and urgent event that could help save more PAH patients.

**Figure:**
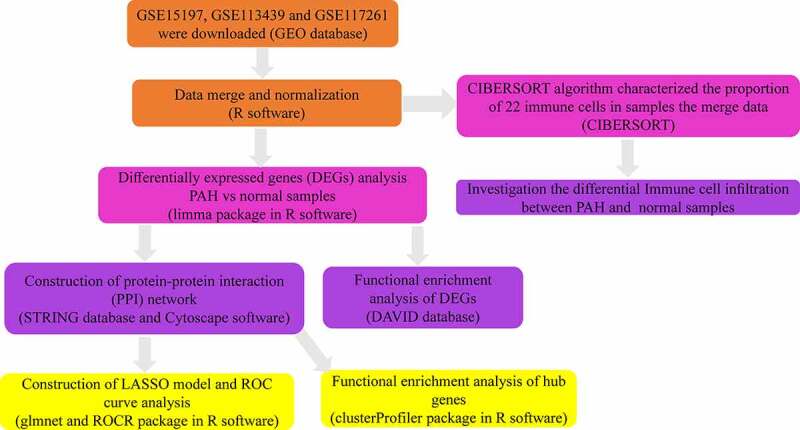

Data mining has been used in a variety of genomic analyses, including genomics, transcriptomes, and epigenetics. Gene chip technology combined with bioinformatics analysis can provide a new and effective method to explore the molecular mechanisms of various diseases through a comprehensive analysis of potential changes in gene expression between abnormal and paired normal tissues. CIBERSORT is a R/Web-based tool that can be applied to deconvolve the gene expression profiles of human immune cell subtypes based on linear support vector regression. The CIBERSORT analysis tool can use standardized gene expression data to estimate the proportion of 22 types of immune cell components in different samples [6]. It has the advantages of high resolution and the ability to simultaneously quantify multiple types of immune cells [6,7]. The pathogenesis of PAH is not well understood. Although some studies have shown that chronic inflammation can cause PAH [8], and there are a few studies on gene expression and immune cells in the big data related to PAH.

**Figure 1.**
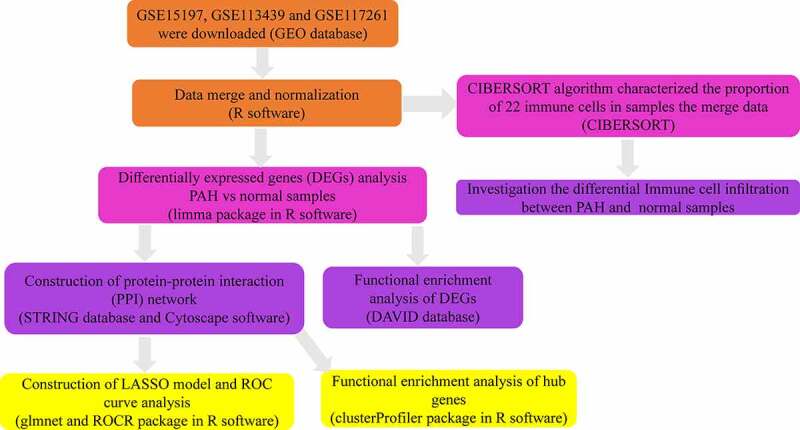
The workflow of this study

In the present study, we re-analyzed the GSE15197, GSE113439, and GSE117261 datasets previously reported by Rajkumar et al. [9], Mura et al. [10], and Stearman et al. [11]. Three microarray mRNA expression datasets were combined, and differentially expressed genes (DEGs) were obtained. Functional enrichment analyses and construction of the protein-protein interaction (PPI) network of DEGs were performed using the STRING and DAVID databases, respectively. The diagnostic value of hub gene expression in PAH was also analyzed. Finally, the infiltration of immune cells in PAH was analyzed using the CIBERSORT algorithm. Figure 1 shows the workflow of the study (Figure 1). We intend to use the information of PAH patients in the GEO database for bioinformatics analysis to identify diagnostic markers and target genes for treatment so as to reduce the harm caused by invasive diagnostic techniques and reduce the side effects caused by nonspecific treatments.

## 2. Materials and methods

### 2.1 Microarray data acquisition

The Gene Expression Omnibus (GEO) (https://www.ncbi.nlm.nih.gov/geo/) is a database that stores chips, second-generation sequencing, and high-throughput sequencing data [12,13]. Gene expression data submitted by the research institutions were included in the GEO database. Three GEO series (GSE15197, GSE113439, and GSE117261) were chosen in our study based on the following selection criteria: (a) keywords of ‘pulmonary artery hypertension (PAH)’ or ‘pulmonary hypertension (PH)’; (b) inclusion of gene expression data of PAH and normal lung tissue samples with the same GEO platform; (c) excluding other diseases except PAH and normal tissues, such as pulmonary fibrosis or interstitial pneumonia; (d) datasets containing a minimum of 10 PAH and normal tissue samples and inclusion of > 5000 genes in the GEO platform. Three mRNA expression data (GSE15197, GSE113439, and GSE117261), after normalization and log2 transformation, were obtained from GEO. GSE15197 was tested on the GPL6480 platform containing gene expression information from 18 PAH lung specimens and 13 normal lung specimens. GSE113439 and GSE117261 were both based on the GPL6244 platform containing 15 PAH lung specimens, 11 normal lung specimens, 58 PAH lung specimens, and 25 normal lung specimens, respectively. All the samples came from different individuals and did not match with each other. Table 1 shows detailed information on the three mRNA expression datasets (Table 1).

**Table 1.** Details of three GEO datasets

Dataset	Tissue	Platform	PAH	Normal	Reference (PMID)
GSE15197	lung	GPL6480	18	13	20,081,107
GSE113439	lung	GPL6244	15	11	30,963,672
GSE117261	lung	GPL6244	58	25	30,562,042

Note: GEO, Gene Expression Omnibus; PAH, Pulmonary arterial hypertension

### 2.2 Data processing

After these three microarray expression matrices were downloaded, R software (version 3.6.3) was used to convert the probe names into gene symbols [14]. The probes were mapped to their respective gene symbol identifiers based on their probe annotation files, and probes annotated to the same gene symbol identifier were aggregated by their mean value [15,16]. The three datasets were integrated as one, and the ‘sva’ package in R software was applied to eliminate batch effects [17].

### 2.3 Screening of DEGs

The DEGs between PAH lung specimens and normal lung specimens were screened out via the ‘limma’ package in R software (version 3.6.3) [18]. The threshold of DEGs was set as |log_2_ fold change (FC)| > 0.5, and *P_adj_*-value < 0.05 [19,20].

### 2.4 Functional analysis of DEGs

The DAVID database (https://david.ncifcrf.gov/) is a biological information database that integrates biological data and analysis tools to provide systematic and comprehensive annotated biological function information for large-scale gene or protein lists to help users extract biological information from them [21,22]. To further explore the biological function of DEGs in PAH, functional enrichment analyses, including Gene Ontology (GO) and Kyoto Encyclopedia of Genes and Genomes (KEGG) enrichment analysis, were performed based on the DAVID database. GO breaks down the function of genes into three categories, including biological process (BP), cellular component (CC), and Molecular Function (MF), and based on these three aspects, we will get the gene annotation information [23]. KEGG enrichment analysis can help researchers understand the signaling pathways that DEGs are involved in [24]. Statistical significance was set at P < 0.05.

### 2.5 Construction of PPI network and module analysis

The study of the interaction network between proteins helps to mine core regulatory genes. At present, there are many databases of protein interactions, among which the Search Tool for the Retrieval of Interacting Genes (STRING) database (http://string-db.org/) is the one with the highest species coverage and the largest interaction information [25]. In this study, a PPI network of DEGs was built based on a minimum interaction value of >0.4. Next, the PPI network was uploaded to Cytoscape software (version 3.7.2) for visualization [26]. Then, the Molecular Complex Detection (MCODE) plug-in Cytoscape software was applied to identify the module in the PPI network with the threshold as flow: the degree cutoff was 2, the node score cutoff was 0.2, the k-core was 6, and the max. depth was100. Further, the GO and KEGG analysis were performed for the genes in the module of the PPI network via ‘clusterProfiler’ package in R software. Statistical significance was set at P < 0.05.

### 2.6 Construction of LASSO model and receiver operating characteristic (ROC) curve analysis

The least absolute shrinkage and selection operator (LASSO) has a strong predictive value and low correlation and is applied to select the best features for high-dimensional data [27]. To distinguish PAH from control, the ‘glmnet’ package in R software was used to construct LASSO model according to the expression profile of hub genes and the diagnosis of the 140 samples. According to the binary output variable in the processed data, we used a binomial distribution variable in the LASSO classification as well as the lambda value with the smallest average error in order to build the model with decent performance but the least number of variables. The expression levels of the hub genes and the diagnosis of the 91 samples were obtained from the probe-matched matrix file. The drawing of the receiver operating characteristic (ROC) curves and the calculation of the area under the curve (AUC) were conducted by the ‘ROCR’ package in R, and the samples were randomly assigned to the training or testing cohort in an approximately 7:3 ratio. Thus, we investigated the feasibility of the hub genes for prediction using the AUC value. An area under the curve (AUC) >0.9 indicated a good diagnostic value [28–30].

### 2.7 Immune cell inﬁltration analysis

The CIBERSORT algorithm was applied to evaluate the proportions of 22 subtypes of inﬁltrating immune cells based on the normalized gene expression data from 91PAH lung specimens and 49 normal lung specimens obtained previously [6]. CIBERSORT is a deconvolution algorithm that contains gene expression reference values from a signature matrix of 547 genes in 22 types of immune cells [6]. The gene expression matrix was uploaded to the CIBERSORT online website (https://cibersort.stanford.edu), and the default signature matrix was set as 1000 permutations, and the samples with *P*-value < 0.05 were significant [31]. The *P*-value of CIBERSORT reflected the statistical significance of the deconvolution results over all cell subsets and was used to filter out deconvolution with less significant fitting accuracy [32]. The difference in immune cell infiltration between PAH lung specimens and normal lung specimens was assessed, and the signiﬁcant immune cells between PAH lung specimens and normal lung specimens were screened using the Wilcoxon test at *P* < 0.05.

### 2.8 Statistics analysis

Categorical variables were presented as percentages, while normally distributed continuous variables were presented as the mean ± standard deviation (SD). The moderate t-test was used for screening DEGs [33]; GO and KEGG annotation enrichments were analyzed using Fisher’s exact test [34]. Immune cell analysis was performed using Wilcoxon’s test. R software (version 3.6.3) was used to perform all statistical analyses and image visualization.

## 3. Results

We intend to use the information of PAH patients in the GEO database for bioinformatics analysis to identify diagnostic markers and target genes for treatment so as to reduce the harm caused by invasive diagnostic techniques and reduce the side effects of nonspecific treatments. We screened out the important target genes associated with PAH by comparing the differences in gene expression profiles between lung samples of PAH and their normal samples. Total 182 DEGs and 15 hub genes were identified, and their functional enrichment analyses were performed. These 15 hub genes are involved in multiple immune responses and immune cell chemotaxis. Meanwhile, a 7-gene-based model was constructed and showed that the diagnostic value of seven genes (S100A8, CD14, ITGAM, C5, CSF3R, PPBP, and CCL21) in distinguishing PAH tissues from normal samples were excellent. Furthermore, we applied the CIBERSORT algorithm to probe immune cell infiltration in PAH. The results showed that the immune cell infiltration of PAH samples was significantly different from that of the normal samples.

### 3.1 Identification of DEGs in PAH

**Figure 2.**
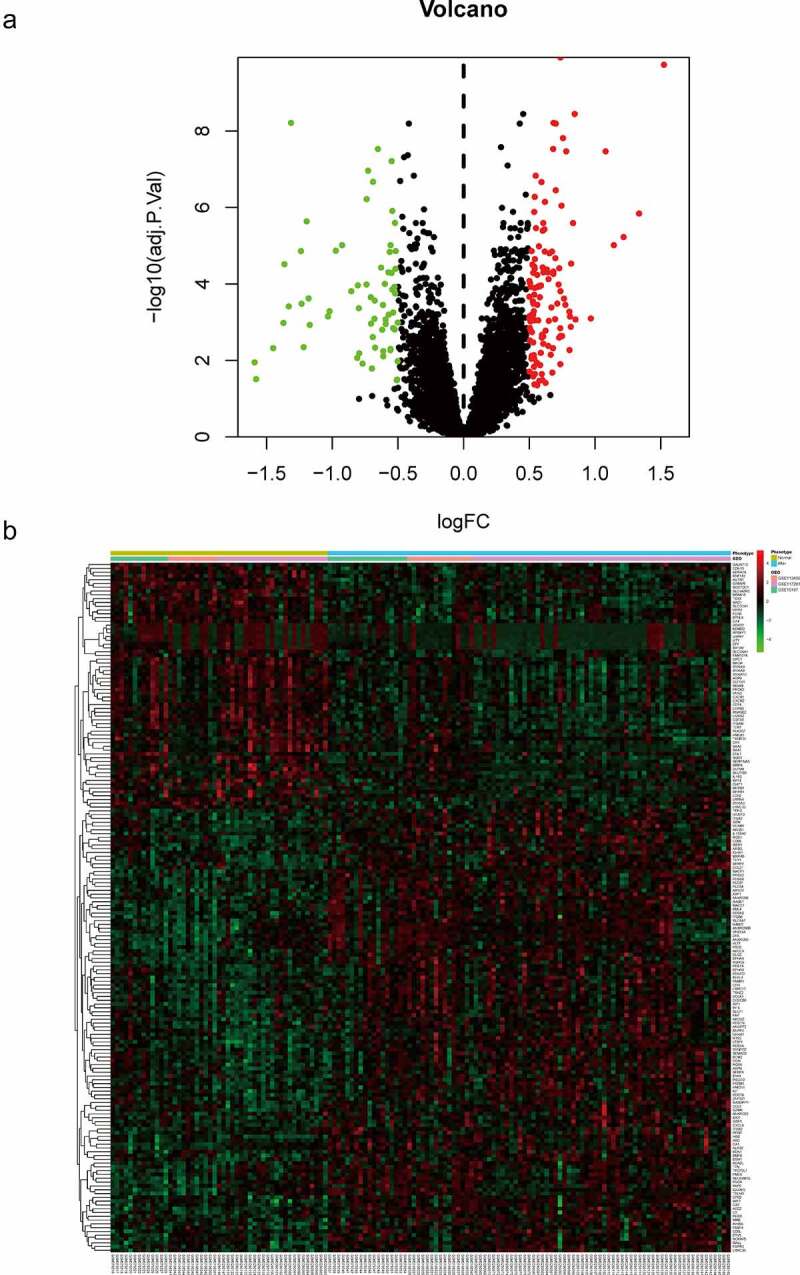
Identification of DEGs from three mRNA expression datasets. (a) Volcano plot of three mRNA expression datasets after integrated as one via R software. log FC, log2 Fold Change. (b) Heatmap of differentially expressed gene expression. The heatmap was generated using pheatmap package in R. The expression profiles greater than the mean are colored in red and those below the mean are colored in green. PAH, Pulmonary arterial hypertension

In our study, 182 DEGs were identified between PAH lung specimens and normal lung specimens. Among them, 117 were upregulated (log_2_ FC>0.5) and 65 were downregulated (log_2_ FC< −0.5) (Table 2). The volcano plot and heatmap of gene expression are shown in Figures 2A and 2B.

**Table 2.** Screening DEGs in PAH by integrated analysis of microarray

DEGs	Gene names
Up-regulated	LTBP1, HBB, ACE2, SECISBP2L, PDE4D, ABCC9, PDE3A, TSHZ2, WIF1, DLG2, ITGB6, PDE7B, FREM1, EPHA4, MACC1, MALL, POSTN, IGF1, HIVEP2, N4BP2, ZFX, PLCB1, SFRP2, PI15, KLHL4, MACF1, PDE1A, PDE8B, ABCG2, ACADL, PREX2, CA1, PLCB4, IQGAP2, XAF1, ANKRD36B, FGFR2, INHBA, RGS5, TXLNG, ECM2, NT5E, ETV5, RASEF, LRRC36, VPS13A, FGD4, GEM, ANKRD36, MXRA5, CFH, ZNF521, CA2, C5, PAMR1, BMP6, GFRA1, RSPO3, THY1, PIEZO2, CCL21, DCLK1, ANKRD50, ALAS2, GBP5, SLC4A7, OGN, SULF1, NR1D2, SYNPO2, RGS1, ASPN, EML4, TFPI2, VCAM1, KIT, WEE1, ABCB1, HLTF, ANGPT2, RASGRP1, ITGB3, PSD3, CCL5, HMCN1, ITGA2, CCDC80, IL13 RA2, EPHA3, FABP4, HBD, CD5L, LRRC17, PHEX, GZMK, ENPP2, ESM1, PDGFD, TTN, MME, TFCP2L1, CD69, EYA4, NCKAP5, CXCL9, EDN1, SEMA3D, PKP2, IDO1, FAP, CPB2, ANKRD22, FMO5, SFRP4, PPBP, AREG, IGHA1
Down-regulated	RNASE2, CSF3R, GIMAP6, ADRA1A, LILRA2, GLT1D1, ITGAM, MGAM, NKD1, TBX3, S100A9, S100A8, LILRB2, SOSTDC1, CD14, SAA2, NQO1, QPCT, TLR8, SLC9A3R2, KRT4, CXCR1, AQP9, AGTR1, GALNT13, SLCO4A1, RNF182, VNN2, S100A12, S100A3, BPIFA1, SULT1B1, USP9Y, ZFY, IL1R2, SLCO2A1, LRRC32, SAA1, BTNL9, TXNRD1, MNDA, UTY, MS4A15, CR1, EIF1AY, CDH13, LRRN4, CXCR2, PROK2, KDM5D, VIPR1, BPIFB1, CHL1, CA4, SERPINA3, CHIT1, LCN2, MMP8, FAM107A, DDX3Y, OLFM4, FCN3, RPS4Y1, PLA2G7, HMOX1

DEGs, differentially expressed genes; PAH, Pulmonary arterial hypertension.

### 3.2 Function analysis of DEGs

**Figure 3.**
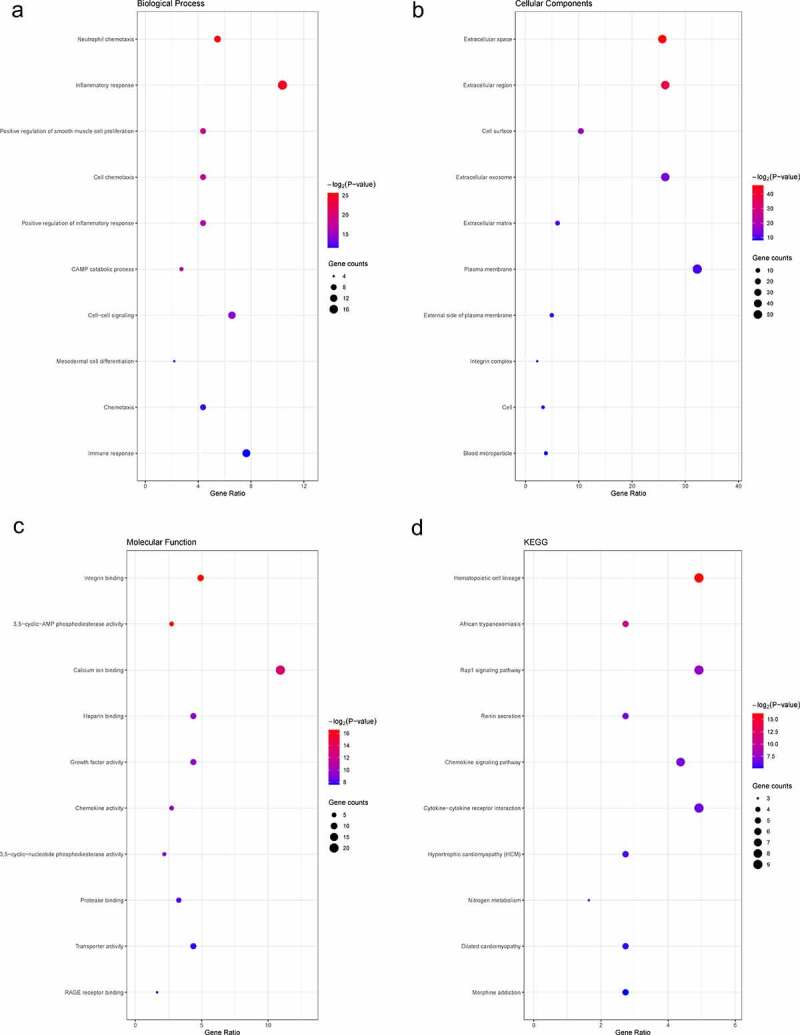
Top 10 enriched GO terms and top 10 KEGG pathways of differentially expressed genes. (A‑C) GO term enrichment analysis for (a) biological process, (b) molecular function, (c) cellular component. (d) KEGG pathway analysis. Node size represents gene ratio; node color represents *P*-value. GO, gene ontology; KEGG, Kyoto Encyclopedia of Genes and Genomes

To explore the function of 182 DEGs in PAH, GO analysis of these 182 DEGs was performed using the DAVID database (Table S1). The top five GO terms are shown in Table 3, and the top ten GO terms are shown in Figure3A-3C according to the *P*-value. In BP analysis, DEGs mainly participated in neutrophil chemotaxis, inflammatory response, positive regulation of smooth muscle cell proliferation, cell chemotaxis, and positive regulation of inflammatory response. In CC analysis, DEGs significantly participated in the extracellular space, extracellular region, cell surface, extracellular exosome, and extracellular matrix. MF analysis showed that DEGs significantly participated in integrin binding, 3ʹ,5ʹ-cyclic-AMP phosphodiesterase activity, calcium ion binding, heparin-binding, and growth factor activity. After uploading the 182 DEGs to the DAVID database, KEGG analysis was performed to explore the pathways of these 182 DEGs (Table S2). The top ten KEGG terms of DEGs based on the *P*-value are shown in Table 4 and Figure 3D. As shown, these DEGs were mainly enriched in Hematopoietic cell lineage, African trypanosomiasis, Rap1 signaling pathway, Renin secretion, and Chemokine signaling pathway (Table 4 and Figure 3D).

**Table 3.** GO analysis of DEGs in PAH

Category	Term	Count	*P*-value	FDR
BP	neutrophil chemotaxis	10	1.94E-08	2.62E-05
BP	inflammatory response	19	5.34E-08	3.61E-05
BP	positive regulation of smooth muscle cell proliferation	8	2.28E-06	1.03E-03
BP	cell chemotaxis	8	3.94E-06	1.33E-03
BP	positive regulation of inflammatory response	8	8.61E-06	2.33E-03
CC	extracellular space	47	1.54E-14	2.73E-12
CC	extracellular region	48	2.31E-12	2.04E-10
CC	cell surface	19	4.97E-06	2.93E-04
CC	extracellular exosome	48	6.73E-05	2.98E-03
CC	extracellular matrix	11	5.97E-04	2.11E-02
MF	integrin binding	9	1.04E-05	2.26E-03
MF	3ʹ,5ʹ-cyclic-AMP phosphodiesterase activity	5	1.21E-05	2.26E-03
MF	calcium ion binding	20	1.05E-04	1.31E-02
MF	heparin binding	8	1.15E-03	8.01E-02
MF	growth factor activity	8	1.24E-03	8.01E-02

Note: GO, Gene Ontology; DEGs, differentially expressed genes; PAH, Pulmonary arterial hypertension; BP, biological process; CC, cellular component; MF, molecule function; FDR, false discovery rate

**Table 4.** KEGG enrichment analysis of DEGs in PAH

Category	Term	Count	*P*-value	FDR
hsa04640	Hematopoietic cell lineage	9	1.33E-05	1.83E-03
hsa04060	African trypanosomiasis	5	7.71E-04	5.28E-02
hsa05418	Rap1 signaling pathway	9	5.22E-03	2.39E-01
hsa04061	Renin secretion	5	8.86E-03	2.60E-01
hsa05144	Chemokine signaling pathway	8	9.48E-03	2.60E-01
hsa04614	Cytokine-cytokine receptor interaction	9	1.22E-02	2.78E-01
hsa04657	Hypertrophic cardiomyopathy	5	1.74E-02	3.33E-01
hsa04062	Nitrogen metabolism	3	1.94E-02	3.33E-01
hsa04064	Dilated cardiomyopathy	5	2.22E-02	3.38E-01
hsa05143	Morphine addiction	5	2.88E-02	3.91E-01

Note: KEGG, Kyoto Encyclopedia of Genes and Genomes; DEGs, differentially expressed genes; PAH, Pulmonary arterial hypertension; FDR, false discovery rate

### 3.3 Construction of PPI network and hub gene analysis

**Figure 4.**
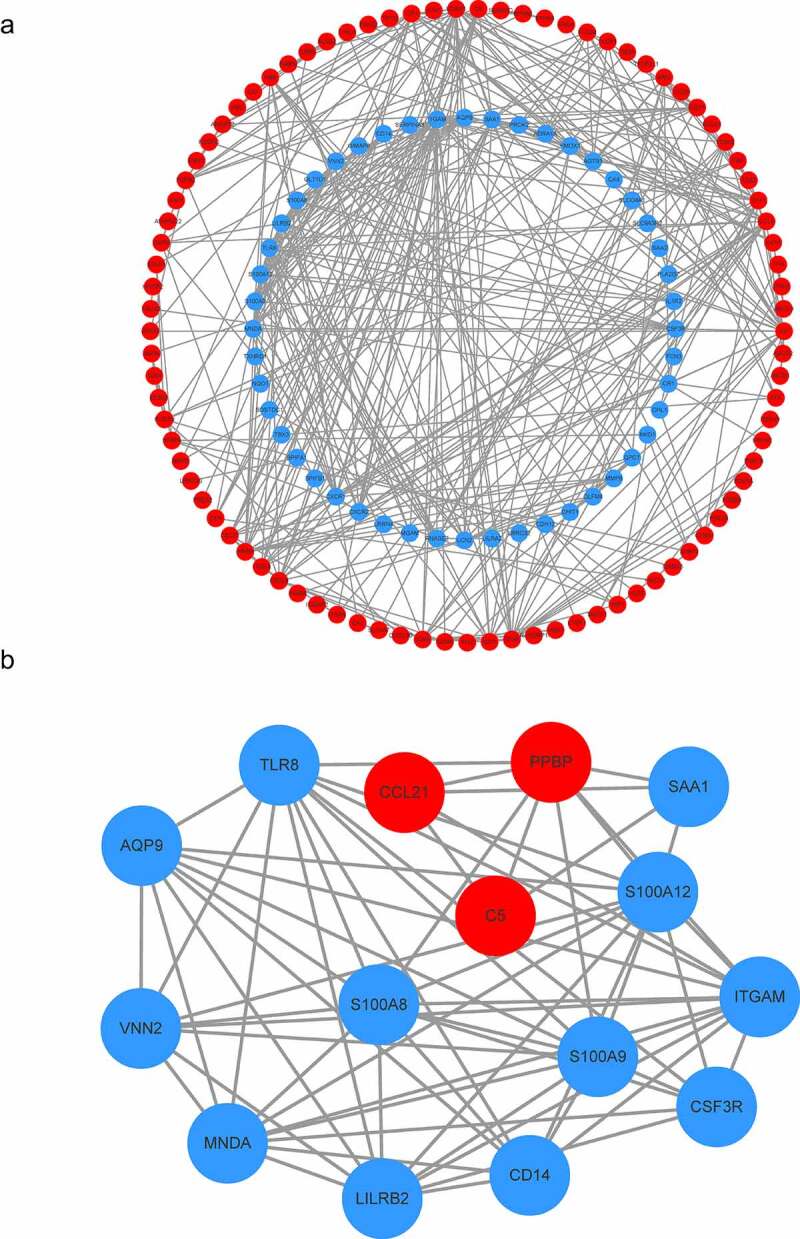
Construction of the PPI network. (a) The nodes represent proteins, and the edges represent the interaction of proteins, while blue and red circles indicate downregulated and upregulated DEGs, respectively. (b) The only one module in the PPI network. The nodes represent proteins, and the edges represent the interaction of proteins, while blue and red circles indicate downregulated and upregulated DEGs, respectively

**Figure 5.**
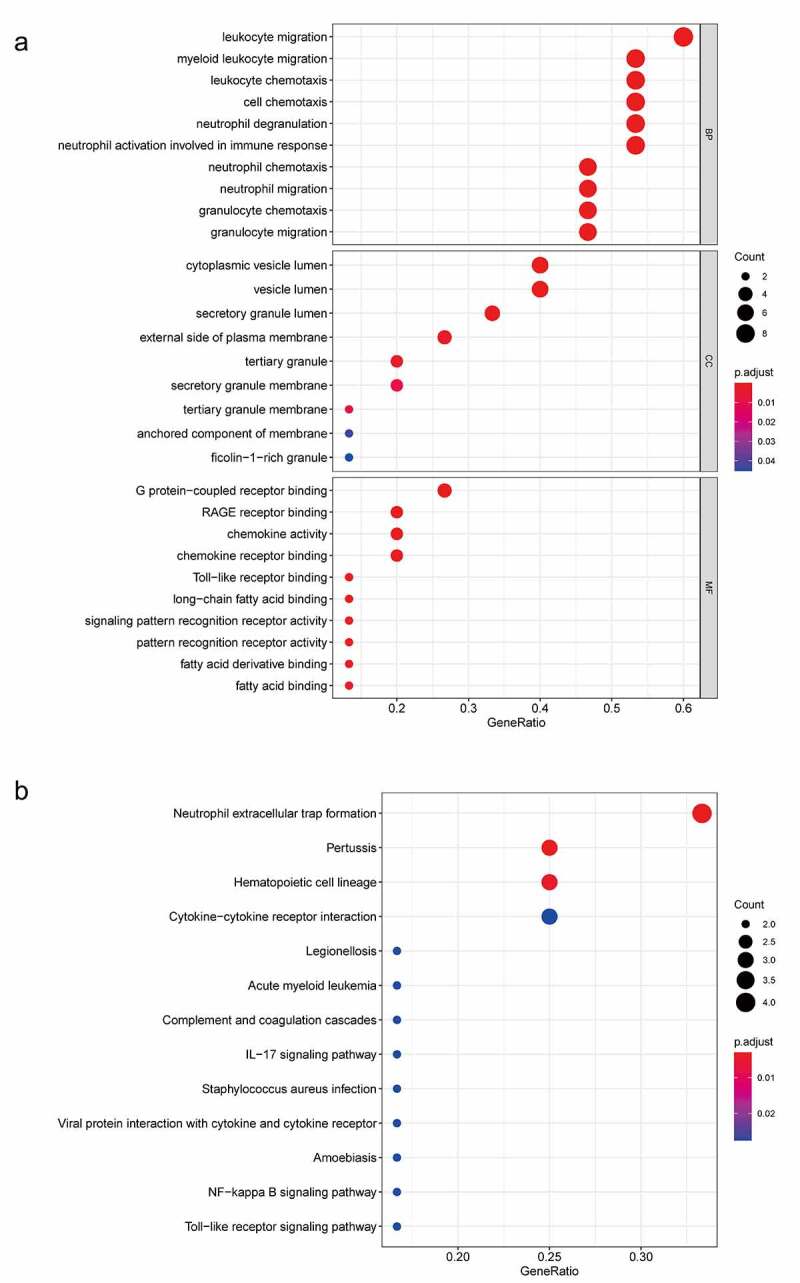
GO and KEGG analyses of module genes. (a) GO term enrichment analysis of module genes. (b) KEGG pathway analysis of module genes. Node size represents gene ratio; node color represents *P_adj_*-value. GO, gene ontology; KEGG, Kyoto Encyclopedia of Genes and Genomes

The STRING database and Cytoscape software were used to establish the PPI network of the DEGs. A PPI network containing 137 genes and 417 edges was constructed (Figure 4A). In the PPI network, the average node degree was 4.77, and the average local clustering coefficient was 0.443. Among these 182 genes, only one module (including 15 genes) was identified by the MCODE plug-in (Figure 4B). Further, function analysis was performed for DEGs in the module with a *P_adj_*-value < 0.05. These 15 hub genes were significantly related to immune system function, such as neutrophil chemotaxis, myeloid leukocyte migration, neutrophil migration, cell chemotaxis, neutrophil extracellular trap formation, IL-17 signaling pathway, Toll-like receptor signaling pathway, and NF-κB signaling pathway (Figure 5 and Table S3).

### 3.4 Exploring candidate biomarkers by lasso regression and receiver operating characteristic curves

**Figure 6.**
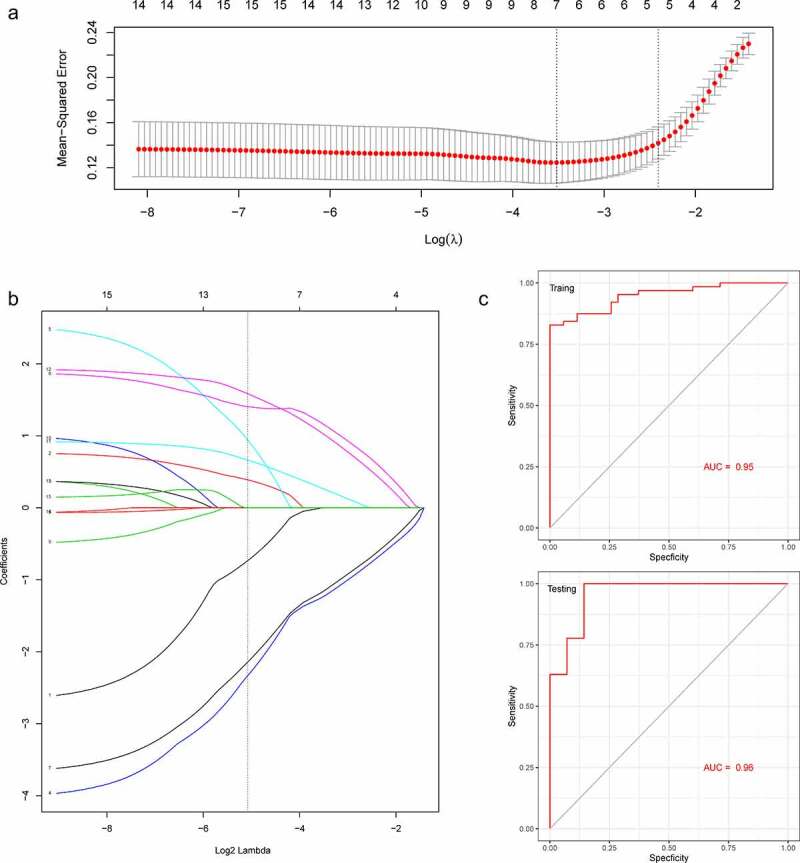
A model for predicting PAH. (a) LASSO model. (b) ROC curves analysis of training set. (c) ROC curves analysis of test set. AUC, area under the curve. PAH, Pulmonary arterial hypertension

To select the best biomarkers of PAH, the 15 hub genes were further analyzed. The LASSO regression method was used to identify seven potential biomarkers (Figure 6A, 6B) with coefficients of −0.0017, −0.0298, −0.1630, 0.1779, −0.1700, 0.0258, and 0.1532 for S100A8, CD14, ITGAM, C5, CSF3R, PPBP, and CCL21, respectively. ROC curve analysis was used to evaluate the ability of the LASSO model to distinguish PAH in the training and testing sets. ROC curve analysis (Figure 6C, 6D) indicated that the AUC of the 7-gene-based model was 0.95, in the training set and 0.96, in the testing set, suggesting that these seven genes have a good diagnostic value for distinguishing PAH from normal controls.

### 3.5 Immune cell inﬁltration analysis

**Figure 7.**
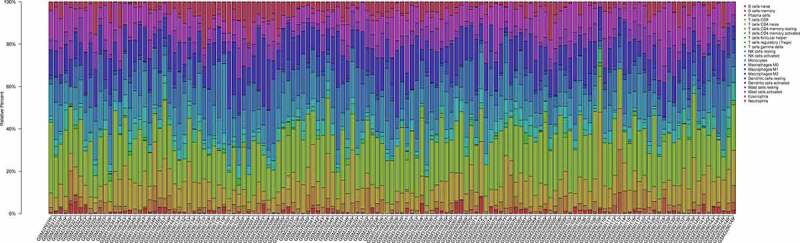
The bar plot visualizing the relative percent of 22 immune cell in each sample. Different colors represent different types of immune cells

**Figure 8.**
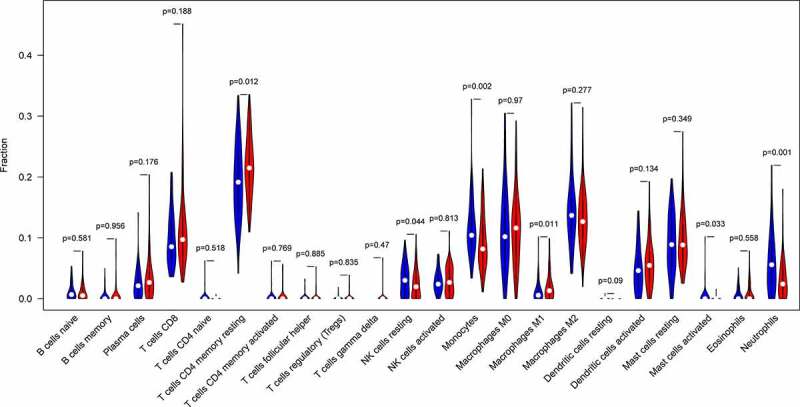
The difference of immune infiltration between PAH samples and normal control samples. Blue, normal controls group; Red, PAH group. PAH, Pulmonary arterial hypertension

Ninety PAH and 49 normal control samples that matched the requirements of CIBERSORT *P*-value < 0.05 were filtered out. The CIBERSORT algorithm was applied to investigate the relative proportion of the 22 types of immune cells in 90 PAH samples and 49 normal control samples (Figure 7). The proportions of T cells CD4 memory resting (*P* = 0.012) and Macrophages M1 (*P* = 0.011) in PAH samples were significantly lower than those in normal control samples (Figure 8). However, the proportion of NK cells resting (*P* = 0.044), Monocytes (*P* = 0.002), Mast cells activated (*P* = 0.033), and Neutrophils (*P* = 0.001) in PAH samples were significantly higher than those in normal control samples (Figure 8).

## 4. Discussion

PAH is defined as a type of chronic progressive malignant pulmonary vascular disease and has similar pathological characteristics to cancer, such as resistance to apoptosis, metabolic changes, and growth factor receptor overexpression. The hemodynamic criteria of PAH are as follows: mean pulmonary artery pressure (mPAP) ≥ 25 mmHg (1 mmHg = 0.133kPa) measured by sea level, resting time, and right cardiac catheterization [35]. According to the 6th World Symposium on Pulmonary Hypertension (WSPH) recommendation, an mPAP ≥ 20 mmHg with a pulmonary vascular resistance (PVR) ≥ 3 Wood units was defined as PAH [36]. It has been reported that TLR3 is involved in endothelial cell apoptosis and pulmonary vascular remodeling and may be a therapeutic target for PAH [37]. A recent study found that treatment with inhaled treprostinil improved exercise performance and reduced NT-proBNP levels in patients with interstitial pulmonary disease due to PAH [38]. Although research on PAH has increased in recent years, the pathogenesis of PAH is still unclear, and the therapeutic effect is unsatisfactory.

In the present study, we screened out the important target genes associated with PAH by comparing the differences in gene expression profiles between lung samples of PAH and their normal samples. In our study, 182 DEGs and 15 hub genes were identified, and functional enrichment analyses were performed. These 15 hub genes are involved in multiple immune responses and immune cell chemotaxis. Furthermore, we applied the CIBERSORT algorithm to probe immune cell infiltration in PAH. The results showed that the immune cell infiltration of PAH samples was significantly different from that of the normal samples.

Establishing the PPI network has been verified to be helpful in the analysis of a disease because all the genes would be grouped and organized in the PPI network according to their interaction [39]. In the present study, we established a PPI network and 15 hub genes, including S100A8, VNN2, CD14, ITGAM, AQP9, C5, CSF3R, SAA1, MNDA, S100A9, PPBP, CCL21, S100A12, TLR8, and LILRB2. A 7-gene-based model was constructed and showed that the diagnostic value of seven genes (S100A8, CD14, ITGAM, C5, CSF3R, PPBP, and CCL21) in distinguishing PAH tissues from normal samples was excellent. S100A8 and S100A9 are the main proteins of peripheral blood mononuclear cells and neutrophils, also known as myeloid-related proteins (MRPs) 8 and 14, or calgranulins A and B [40]. S100A8 and S100A9 are often combined by non-covalent bonds to form the S100A8/A9 heterodimer calprotectin to perform its function [41]. When S100A8/9 is secreted, it binds to a variety of protein receptors on different types of cells, of which the receptors of advanced glycation endproducts (RAGE) and Toll-like receptor 4 (TLR4) are particularly important. Previous studies have suggested that RAGE may be critical in PAH by participating in the etiology of PAH [42,43]. The S100A8/A9 heterodimer may induce endothelial cell (EC) dysfunction in the following ways: by promoting inflammatory responses by increasing the expression of inflammatory cytokines, including IL-6, IL-8, IL-10, IFNγ, VCAM-1, and ICAM-1 in ECs, which are involved in phenotypic transformation and proliferation of vascular smooth muscle cells [44–46]. These studies provide the basis for the involvement of S100A8 and S100A9 in the pathophysiology of PAH. CD14 was known as a receptor for bacterial endotoxin (LPS) in 1990 and was initially identified as a marker of differentiation on the surface of monocytes and macrophages [47]. Studies have identified that CD14 plays a critical role in inflammatory diseases, metabolic diseases, tumors, and other diseases [48]. CD14 promotes atherosclerosis by regulating the function of vascular endothelial cells and smooth muscle cells [48]. These results suggest that CD14 may be involved in the pathophysiological process of PAH by regulating the inflammatory response, vascular endothelial cells, and vascular smooth muscle cells. ITGAM, also called CD11b, is a marker of leukocytes and is closely associated with inflammation in PAH [49,50]. At present, the most studied PAH-related genes are BMPR2, ACVRL1, CAV1, SERT, and KCNK3 [51–54]. Few studies have been conducted on the link between the key genes screened in this study and PAH, which may be new genes for the pathogenesis of PAH. These genes not only provide a suggestion for future research on the pathogenesis of PAH but may also be potential molecular diagnostic markers of PAH.

According to the functional enrichment analysis, 15 hub genes were mainly enriched in neutrophil chemotaxis, myeloid leukocyte migration, neutrophil migration, cell chemotaxis, Neutrophil extracellular trap formation, IL-17 signaling pathway, Toll-like receptor signaling pathway, and NF-κB signaling pathway. These results suggest that inflammatory and immune responses are vital for the occurrence of PAH, which is consistent with previous studies. The NF-κB signaling pathway is activated in the PAH model, and sevoflurane may inhibit the activation of the NF-κB signaling pathway by downregulating the levels of p-IκB, p-p65, and p65, thereby reducing pulmonary fibrosis and preventing PAH [55]. It has been reported that inhibition of the TLR/NF-κB pathway may also provide potential clinical significance in patients with PAH, including the reduction of inflammatory/immune responses and pulmonary vascular remodeling [56]. Studies have shown that IL-1β, IL-6, and TNF-α are related to pulmonary vascular remodeling in PAH [57]. The TLR family is a pattern recognition receptor that recognizes microbial fragments and activates downstream NF-κB pathways. It has been found that decreased TLR3 expression contributes to endothelial cell apoptosis and pulmonary vascular remodeling [37]. These studies provide evidence for the role of inflammatory and immune responses in the pathophysiological process of PAH.

Immune dysregulation has been associated with various diseases, including PAH [58]. NK cells play an important role in preventing endothelial injury and regulating vascular remodeling and regeneration, and NK cell defects may be related to the increased risk of death in patients with PAH [59]. In this study, we found that NK cells resting in PAH samples were significantly higher than those in normal control samples. Therefore, we consider that NK cells are important for the occurrence and development of PAH, but further studies are needed to determine the exact pattern of NK cells in patients with PAH. The main pathophysiological process of PAH is pulmonary vascular remodeling, and studies have shown that mast cells may be involved in the pathophysiological process of pulmonary vascular remodeling [58]. Mast cells may be involved in the angiogenesis of pulmonary hypertension by secreting vascular endothelial growth factor [60,61]. Targeting mast cells against several causes of PAH may help improve vascular remodeling, according to the results from animal models. In this study, we found that mast cells activated in PAH samples were significantly higher than those in normal control samples. Therefore, we consider that mast cells are important for the occurrence and development of PAH, but further studies are needed to determine the exact pattern of mast cells in PAH patients. Immune cells play an indispensable role in the process of pulmonary hypertension vessel remodeling. Therefore, attention should be paid to the mechanism of immune cell infiltration in patients with PAH.

## 5. Conclusions

In this study, 182 DEGs and 15 hub genes were identified. Functional enrichment analysis of these genes provides more information for understanding the pathophysiological mechanism of PAH. The CIBERSORT method was used to investigate immune infiltration in PAH and found that there was a difference in the immune infiltration between PAH samples and normal control samples. The relationship between key genes and immune invasion in the occurrence and development of PAH needs to be studied further.

## Supplementary Material

Supplemental Material
